# Human melanopsin forms a pigment maximally sensitive to blue light (*λ*_max_ ≈ 479 nm) supporting activation of G_q__/11_ and G_i/o_ signalling cascades

**DOI:** 10.1098/rspb.2012.2987

**Published:** 2013-05-22

**Authors:** Helena J. Bailes, Robert J. Lucas

**Affiliations:** Faculty of Life Sciences, University of Manchester, Oxford Road, Manchester M13 9PT, UK

**Keywords:** melanopsin, action spectrum, photopigment, G-protein, intrinsically photosensitive retinal ganglion cells

## Abstract

A subset of mammalian retinal ganglion cells expresses an opsin photopigment (melanopsin, Opn4) and is intrinsically photosensitive. The human retina contains melanopsin, but the literature lacks a direct investigation of its spectral sensitivity or G-protein selectivity. Here, we address this deficit by studying physiological responses driven by human melanopsin under heterologous expression in HEK293 cells. Luminescent reporters for common second messenger systems revealed that light induces a high amplitude increase in intracellular calcium and a modest reduction in cAMP in cells expressing human melanopsin, implying that this pigment is able to drive responses via both G_q_ and G_i/o_ class G-proteins. Melanopsins from mouse and amphioxus had a similar profile of G-protein coupling in HEK293 cells, but chicken Opn4m and Opn4x pigments exhibited some G_s_ activity in addition to a strong G_q__/11_ response. An action spectrum for the calcium response in cells expressing human melanopsin had the predicted form for an opsin : vitamin A1 pigment and peaked at 479 nm. The G-protein selectivity and spectral sensitivity of human melanopsin is similar to that previously described for rodents, supporting the utility of such laboratory animals for developing methods of manipulating this system using light or pharmacological agents.

## 1. Introduction

Animal photodetection relies on a class of G-protein-coupled receptors (GPCRs) composed of an apoprotein (opsin) and associated light-sensitive chromophore (a retinaldehyde). These pigments initiate light-dependent intracellular cascades the nature of which are determined by the class of G-protein to which they couple. Across the animal kingdom, there are opsins which activate Gα subunits of the Gα_i/o_ (G_i_) class, leading to a reduction in cyclic nucleotide concentrations [1–3]; Gα_s_ class, increasing in cyclic nucleotide concentrations [4]; and Gα_q_ (Gα_q__/11_) class, stimulating a phospholipase C-dependent cascade leading to a range of physiological events, including changes in intracellular calcium [5]. There are currently no known examples of opsins coupling to a fourth G protein family that primarily activates the Rho A pathway (Gα_12/13_).

The mammalian visual system relies principally on the absorption of light by rod and cone photoreceptors. However, the evidence from the past decade or so has revealed a third class of photoreceptor within the eye; intrinsically photosensitive retinal ganglion cells (ipRGCs), which express their own distinct opsin—melanopsin [6,7]. Melanopsin cells in mammals are specialized for measuring ambient illumination, contributing to visual discrimination [8,9], and driving a wide variety of physiological responses including, but not restricted to: synchronization of circadian clocks to light : dark cycles, regulation of pupil size, modulation of sleep and suppression of pineal melatonin production [10–14].

Melanopsin orthologues have been described in every major vertebrate class, as well as the basal cephalochordate, amphioxus [15]. Based upon a comparison of amino acid sequences, the melanopsins have been considered to be both structurally and phylogenetically more closely related to the rhabdomeric opsins found in invertebrate ocular photoreceptors than vertebrate rod and cone pigments [15,16]. Rhabdomeric opsins couple to a Gα_q__/11_ protein triggering an IP3/DAG transduction pathway, and there is evidence that this is also the case for melanopsin [17,18]. In mammals, most such evidence relates to melanopsins from mouse and rat, with data showing that rodent ipRGC light responses include an increase in intracellular calcium and are impaired following inhibition of PLC or TRP channel activity [19–23]. More direct assessments of G-protein coupling have indicated that mouse melanopsin can interact with both G_q__/11_ and G_i_ classes [24,25]. Thus far, G-protein selectivity of primate melanopsin remains unexplored.

The relative responsiveness of a photoreceptor cell to different wavelengths of light (spectral sensitivity) is a fundamental determinant of its sensory capabilities. Determining the spectral sensitivity of mammalian melanopsins has proved challenging. ipRGCs contain this pigment at very low density, precluding direct measurement of spectral absorbance *in situ*. Attempts to measure the absorbance spectrum of mammalian melanopsins purified *in vitro* have provided contradictory findings, with peak spectral absorbance (*λ*_max_) of 424, 467 and 500 nm reported for mouse melanopsin [25–27]. The most consistent data have come from measuring the spectral sensitivity of physiological events elicited by melanopsin. In the case of rodents, this has been achieved by direct measurement of ipRGC light responses, and by recording behavioural/physiological responses in animals lacking rods and cones (and thus relying solely on melanopsin for their photosensitivity). In all such cases, peak sensitivity around 480 nm has been reported [7,28,29], and this is generally accepted to be an accurate reflection of the spectral sensitivity of rodent melanopsins *in situ*. Unfortunately, similar approaches cannot be used to define the spectral sensitivity of human melanopsin, because it is not possible to gain direct access to ipRGCs or to eliminate rod/cone signalling in this species. As a result, the spectral sensitivity of human ipRGCs has thus far been inferred from assessments of physiological responses thought to be downstream of this photoreceptor. The resultant action spectra peak in the short wavelength portion, but *λ*_max_ are quite variable, ranging from 446 to 483 nm [30–33]. A more direct measure of spectral absorbance for human melanopsin would be most valuable in resolving that uncertainty.

We aimed to address the uncertainty regarding the spectral sensitivity and G-protein selectivity of human melanopsin by studying this pigment under heterologous expression in HEK293 cells. Our data are consistent with the hypothesis that human melanopsin shows selectivity for a G_q__/11_-coupled signalling pathway, but provide some evidence of activation of G_i/o_. This was not true for all melanopsins, however, with chicken melanopsins showing coupling to G_s_ in addition to G_q__/11_. We continue to describe the wavelength dependence of the light response elicited by human melanopsin in these cells. The resultant action spectrum has the form predicted for that of an opsin : vitamin A-based pigment with peak sensitivity at 479 nm.

## 2. Material and methods

### (a) Cell culture

HEK293 cells were maintained at 37°C in Dulbecco's modified Eagle's medium, 4.5 g l^−1^ d-glucose, sodium pyruvate and l-glutamine (Sigma) with 10 per cent foetal calf serum (Sigma) and penicillin/streptomycin in a 5 per cent CO_2_ atmosphere.

### (b) Construction of expression vectors

Mammalian expression vectors were constructed using pcDNA3.1 and pcDNA5/FRT/TO (Invitrogen) and the open reading frames of chicken Opn4x, chicken Opn4m, mouse Opn4, amphioxus Opn4, human Opn4, human Rh1 and jellyfish opsin (see the electronic supplementary material).

To make an expression plasmid for a luminescent cAMP reporter, the region for the Glosensor cAMP biosensor was excised from pGlosensor 22F (Promega) and ligated into linearized pcDNA5/FRT/TO. All restriction enzymes were from New England Biolabs (NEB). A luminescent calcium reporter was synthesized using the photoprotein aequorin from *Aequorea Victoria* [34] (genbank accession no. AEVAQ440X; electronic supplementary material).

### (c) Opsin expression analysis using western blotting and immunocytochemistry

HEK293 cells were plated in six well plates and transfected with opsin expressing plasmids using Lipofectamine 2000 (Invitrogen) and incubated overnight with 9-*cis* retinal. Protein was extracted from cells with RIPA lysis buffer, charge separated by electrophoresis and transferred onto PVDF membrane (electronic supplementary material). The membrane was incubated with 1 : 1000 monoclonal mouse rhodopsin (1D4) antibody (AFMA1722, Thermo Scientific Pierce) and goat anti-mouse secondary (Dako) and developed for chemiluminescent detection.

HEK293 cells were also plated onto coverslips and transfected with plasmids before overnight incubation with 9-*cis* retinal. Cells were fixed in 4 per cent paraformaldehyde and incubated with 1 : 1000 1D4 antibody and 1 : 1000 Alexa 488 anti-mouse secondary antibody (Molecular Probes). Photomicrographs were taken using a Delta vision microscope (see the electronic supplementary material).

### (d) Luminescent second messenger assays

HEK293 cells were transiently transfected with reporters and opsins prior to assays and incubated overnight with 9-*cis* retinal (see the electronic supplementary material).

### (e) cAMP increases: G_s_

For measurements of cAMP increases as an indication of G_s_ activity, wells of cells were transfected with pcDNA5/FRT/TO Glo22F and opsin in triplicate. Following transfection and overnight incubation with tetracycline/retinal, cells were incubated with additional 2 mM beetle luciferin for 2 h at room temperature (potassium salt reconstituted in 10 mM HEPES buffer; Promega). Luminescence was measured with a Fluostar Optima plate reader (BMG Labtech, Germany; electronic supplementary material).

### (f) cAMP decreases: G_i/o_

Decreases in cAMP are difficult to measure from baseline cAMP reporter luminescence and so cells were treated with 2 µM forskolin (Sigma-Aldrich) to artificially raise cAMP levels. Pertussis toxin, where used, was added to overnight retinal media at 100 ng ml^−1^ (Tocris Cookson Ltd). Luminescence was measured and then cells were exposed to a camera flash, as above. Plate reader settings were otherwise identical as for cAMP increase assays. The average triplicate value for the maximum forskolin-induced Glosensor RLU immediately following light treatment was used to normalize individual well values of the triplicate that were then averaged to give one set of values per genotype per experiment.

### (g) Ca^2+^ increases: G_q__/11_

G_q__/11_ signalling was assessed using the aequorin reporter (constructed as above). There is a sigmoidal sensitivity relationship between the calcium concentration and the log light released by aequorin, making this a good tool with which to examine changes in calcium concentration without the need for exogenous light [35].

Cells transfected with pcDNA5/FRT/TO mtAeq and melanopsin were incubated with 10 µM Coelenterazine *h* (Biotium) in the dark for 2 h before recording luminescence on a plate reader (see the electronic supplementary material).

### (h) Mouse and human Opn4 action spectra

Cells were transfected as above, but 10 µM 11-*cis* retinal was used to incubate cells overnight (courtesy of Rosalie Crouch and the National Eye Institute, National Institutes of Health, USA). HEK293 cells have some retinaldehyde recycling capability [36,37], leaving the possibility that small amounts of other retinal isoforms may be present in the cell milieu. However, we are confident that the majority response relies upon the exogenous chromophore because cells lacking this treatment gave at most a small melanopsin response. Action spectra were described by constructing irradiance response curves to a 2 s flash of near monochromatic light (Xe Arc source filtered with bandpass and neutral density (ND) filters). Each well was only exposed to one light exposure, with the appearance of different irradiances randomly assigned to wells across the plate. Twenty-four wells within a plate were filled with cells and three wavelengths were measured with each ND setting. The three wavelengths covered within a plate were evenly spread across the spectrum (e.g. plate 1: 420, 458, 580 nm; plate 2: 442, 500, 600 nm). A dummy 96 well plate (black walls clear base) containing 150 µl media/well was used to measure irradiance, as experienced by the cells, using an optical power meter (PM203 Optical Power Meter; Macam Photometrics).

Cell responses to each wavelength at each ND setting were measured on three different occasions. The resultant three independent irradiance response curves for each wavelength were fitted with sigmoidal dose response function (variable slope, minimal asymptote constrained to the average baseline RLU value for each wavelength set). By eye, it was clear that the curves for 580 and 600 nm responses (and in mouse also 568 nm) did not reach saturation point. The maximal response for these curves was therefore set as the saturation point for a shorter wavelength within that plate. Relative sensitivity for each curve was estimated by multiplying the inverse of the irradiance required to drive a 50 per cent response (EC50) by the average EC50 for the wavelength of greatest sensitivity (lowest EC50). The average relative sensitivity and standard error of the mean were plotted against Govardovskii visual templates of pigment spectral sensitivity of varying wavelengths [38], and the sum of least squares was calculated to find the curve with the best fit to our measured data. The peak (*λ*_max_) of this curve was assigned as the *λ*_max_ of mouse or human melanopsin in HEK293 cells. Graphpad Prism was used for all statistical analyses.

## 3. Results

**Figure 1.**
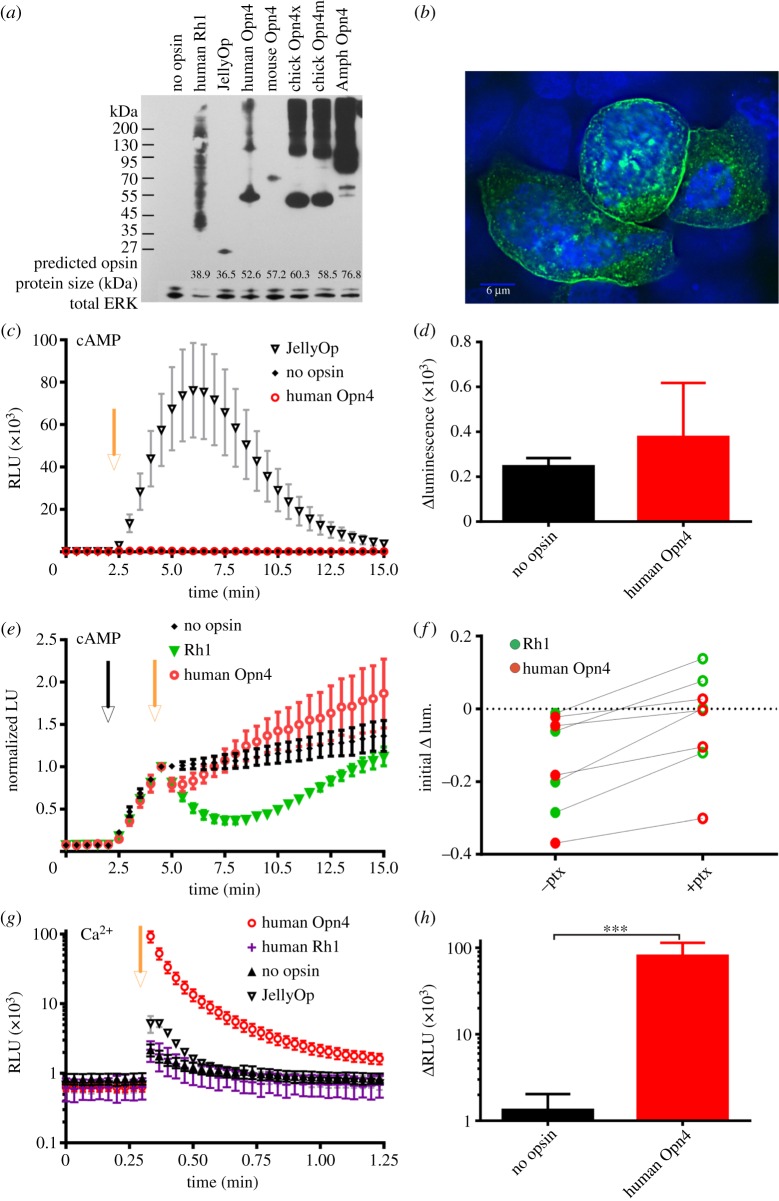
Human melanopsin in HEK293 cells. (*a*) Western blot of opsin protein expressed and extracted from HEK293 cells. Blots were stripped and re-probed with an antibody against total ERK protein to indicate protein amount. Predicted sizes of the opsin proteins as calculated with ExPASy.com are provided at foot of blot. (*b*) Immunocytochemistry photomicrograph showing detection of human melanopsin expressed in HEK293 cells and labelled with a 1D4 antibody (green). Cells are also stained with DAPI, a blue nuclear stain. Melanopsin can be seen mostly localized at the cell membrane and in cytoplasmic inclusions. Scale bar = 6 µm. (*c*) A luminescent biosensor was used to monitor changes in cAMP production. The camera flash (yellow arrow) induced a large increase in cAMP-dependent luminescence indicative of G_s_ activity in cells expressing JellyOp but not those expressing human Opn4. (*d*) In this assay, there was no difference in the change in luminescence following light exposure in cells expressing human Opn4 and no opsin controls (*t*-test *p* > 0.05; *n* ≥ 3). (*e*) Reductions in cAMP as a result of G_i/o_ activity are measured by artificially raising cAMP with forskolin (black arrow) before exposing cells to light (yellow arrow). Human rod opsin (Rh1) induces a prolonged reduction in cAMP production. Luminescence was normalized to values immediately following light exposure (*n* ≥ 6). (*f*) Comparison of the reduction of forskolin-induced cAMP production 1 min following light in human melanopsin or Rh1 expressing cells treated or untreated with 100 ng ml^−1^ pertussis toxin (ptx). A pairwise comparison shows the light-dependent reduction is significantly reduced when treated with ptx, a G_i/o_-specific inhibitor (paired *t*-test; **p < 0.01 both opsins). The inhibition appears greater in Rh1 expressing cells. (*g*) Aequorin luminescence in HEK293 cells expressing opsins as labelled. A camera flash of white light was applied (yellow arrow) and aequorin responses in a 96 well plate reader were monitored. *n* ≥ 4 except human rod opsin (Rh1), *n* = 2. (*h*) There was a significant difference in peak of luminescence immediately following light exposure between no opsin controls and human melanopsin (Opn4; *t*-test ***p < 0.001). All plots show mean ± s.e.m.

Melanopsins from a variety of species could be expressed in HEK293 cells at levels that were easily detectable by western blot and immunocytochemistry (figure 1*a,b*). In the case of human melanopsin, the majority of the protein produced was of expected size for a monomer (approx. 52.6 kDa; figure 1*a*), and much of it was localized to the cell membrane (as expected for opsins), with the remainder in small intracellular inclusions (figure 1*b*).

### (a) Human melanopsin causes no measurable light-dependent increase in cAMP

Opsins capable of activating a G_s_ signalling cascade in this system should support light-dependent increases in cAMP. We tested this prediction using the GloSensor cAMP reporter. Accordingly, we found strong light-dependent increases in luminescent reporter activity in cells expressing JellyOp, a known G_s_-coupled pigment (unpaired *t*-test *p* < 0.01; figure 1*c*). In contrast with JellyOp, however, no significant increase in cAMP following light exposure occurred in human melanopsin-expressing cells, compared with no opsin controls (*t*-test *p* > 0.05; figure 1*d*).

### (b) Human melanopsin triggers some G_i/o_ protein signalling

We next used a modification of the Glosensor cAMP reporter protocol to determine whether melanopsin supported a light-dependent reduction in cAMP. This would be predicted if it were coupling to a G_i_ family signalling cascade. Low basal levels of cAMP in HEK293 cells make reduction in reporter luminescence hard to detect. In order to overcome this problem, cells were first treated with forskolin to elevate cAMP. The success of this strategy was confirmed using human rod opsin, whose native G-protein (G_t_) shares most homology with G_i/o_ proteins. As predicted, light induced a marked and prolonged reduction in cAMP in cells expressing human rod opsin (figure 1*e*). This was not observed in cells expressing the G_s_-coupled Jellyfish opsin (data not shown). Cells expressing human melanopsin also showed a small but reproducible decrease in luminescence in the first minute following light exposure (unpaired *t*-test *p* < 0.01; figure 1*e*) suggesting that this pigment may be capable of interacting with a G_i_ family G-protein. We further tested this hypothesis by treating cells with pertussis toxin, which specifically inhibits G_i/o_ signalling. As predicted, pre-treatment with 100 ng ml^–1^ pertussis toxin for 18 h inhibited this light response in cells containing either melanopsin or rod opsin (figure 1*f*, paired *t*-test *p* < 0.01 for both opsins).

### (c) Human melanopsin causes large light-dependent increases in calcium

GPCR coupling to G_q_ cascades leads to an increase in calcium mobilization predominantly through phospholipase C activation and inositol triphosphate release. We assessed calcium changes in HEK293 cells expressing a luminescent reporter of intracellular calcium (aequorin). An intense flash of light induced a short-lived increase in luminescence even in the absence of opsin expression, which we presume is due to photo-oxidation of the coelenterazine (figure 1*g*). Cells expressing human melanopsin, however, exhibited a much bigger increase in luminescence (unpaired *t*-test, *p* < 0.001, figure 1*h*), peaking at or before the first reading after light exposure and decaying to near baseline levels over the course of a minute. Human melanopsin is therefore capable of inducing significant calcium mobilization in HEK293 cells.

This light-dependent calcium increase matches a prediction of the hypothesis that melanopsin activates a G_q__/11_ cascade in these cells. However, calcium responses can also be observed as a secondary consequence of other G-protein signalling events. In the case of human melanopsin, this seems unlikely. Firstly, although cells expressing the G_s_-coupled JellyOp did also display a light-dependent calcium response, we were unable to detect any G_s_ activity from human melanopsin in this system (figure 1*c*). We of course cannot exclude the possibility that human melanopsin has some G_s_ coupling that falls below the detection limit of our assay, but given the relatively small calcium signal induced by JellyOp, which had a very large G_s_ response, this does not represent a credible explanation for the melanopsin-driven calcium response. Human melanopsin did induce a small G_i/o_-driven decrease in cAMP, but again this appears not to explain the calcium response. Not only was the human melanopsin-driven calcium response insensitive to pertussis toxin (data not shown), but also rod opsin, which strongly activated a G_i/o_ cascade, did not drive a detectable increase in calcium (figure 1*g*).

### (d) G-protein selectivity in melanopsins from other species

**Figure 2.**
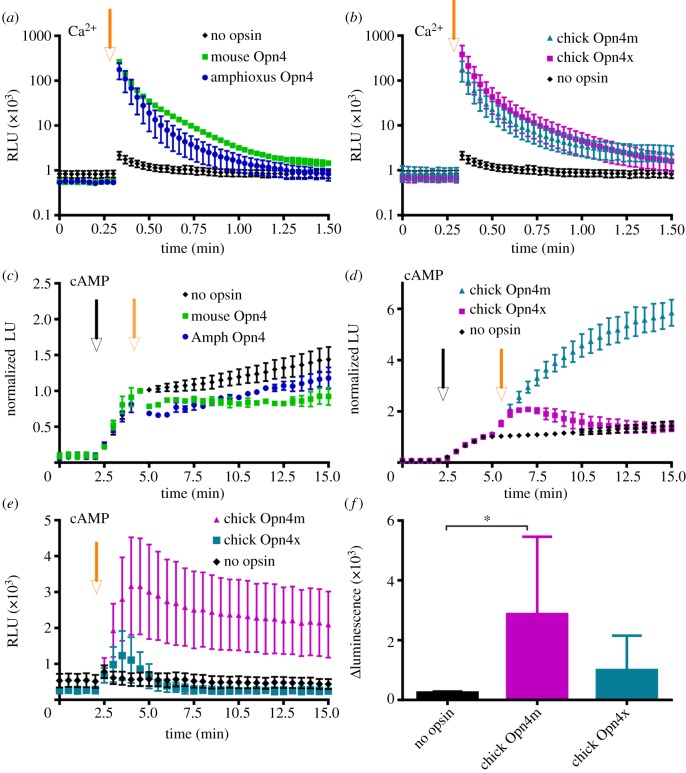
G-protein coupling of melanopsins from other species. G_q__/11_ assays showed robust calcium-dependent increases in aequorin luminescence from mouse and amphioxus Opn4 (*a*) and chicken Opn4m and Opn4x (*b*). (*c*) Amphioxus and mouse Opn4 showed a small inhibition of forskolin (black arrow)-induced luminescence following light exposure (grey arrow) indicative of a minor G_i/o_ response. (*d*) Conversely, both chicken Opn4x and Opn4m cause an increase in cAMP reporter luminescence following forskolin and light exposure. (*e*) This light-dependent increase in cAMP production (indicative of G_s_ coupling) is also measurable when no forskolin is first added. All plots show mean ± s.e.m.; *n* = ≥ 3. (*f*) The maximum difference in cAMP reporter luminescence before and after light exposure is significantly greater in Opn4m but not Opn4x expressing cells compared with when no opsin is expressed (Mann Whitney test *p < 0.05). Mean ± s.e.m., *n* = 4.

We next determined whether calcium and cyclic nucleotide components of GPCR signalling were measurable in melanopsins from other species in this system. HEK293 cells expressing melanopsins from mouse, amphioxus and two separate melanopsin proteins from chicken (Opn4x and Opn4m) all exhibited robust light-induced increases in aequorin luminescence whose amplitude and decay kinetics were similar to those seen from human melanopsin (figure 2*a,b*). Mouse and amphioxus melanopsins also showed a light response in our assay of G_i/o_ activity, with modest decreases in Glosensor luminescence in HEK293 cells treated with forskolin (figure 2*c*). Conversely, both chicken melanopsins showed a large light-dependent *increase* in luminescence in this assay, indicative of enhanced cAMP accumulation (figure 2*d*). We therefore went on to examine light-induced cAMP production in cells not pretreated with forskolin, i.e. a more straightforward measure of G_s_ coupling. Both chicken melanopsins caused an increase in cAMP reporter activity following light, although this was significant and much more prolonged only in cells expressing chicken Opn4m (figure 2*e*,*f*). HEK293 cells expressing either mouse or amphioxus melanopsin did not show any light-induced increase in cAMP in this assay (data not shown).

### (e) Spectral sensitivity of human and mouse melanopsins

**Figure 3.**
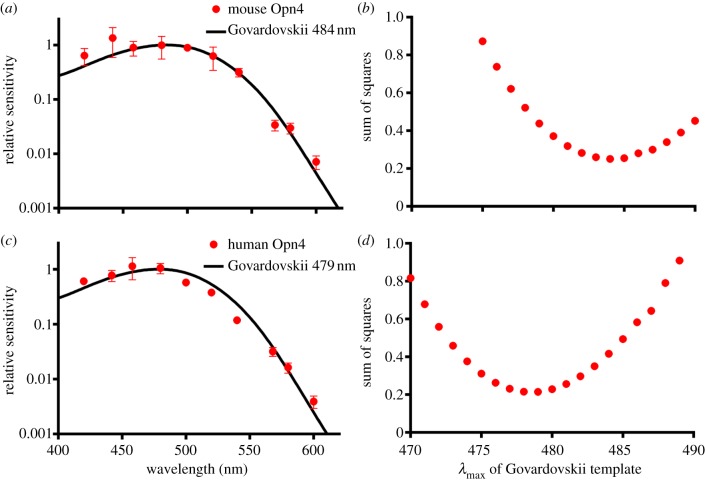
Spectral sensitivity of melanopsin. Action spectra for (*a*) mouse and (*b*) human melanopsins were defined by plotting the relative sensitivity at each wavelength (mean ± s.e.m. of the three replicate irradiance response curves). The action spectra were fit with the predicted absorbance spectrum of opsin : vitamin A-based photopigments with *λ*_max_ between 450 and 550 nm, and the best fit assessed by minimizing sums of squares. Mouse melanopsin best matches a template with *λ*_max_ 484 nm, and human melanospin *λ*_max_ 479 nm. Sum of squares for comparisons between our data and opsin templates with *λ*_max_ between 470 and 490 nm are shown as an indication of the confidence of these estimates for (*c*) mouse and (*d*) human melanopsin.

We next set out to explore the spectral sensitivity of the robust aequorin light response as a method of determining the spectral sensitivity of human melanopsin. To this end, we described irradiance response curves for near monochromatic stimuli spanning the visible spectrum (see the electronic supplementary material, figure S1). To validate this approach, we first applied it to HEK293 cells expressing mouse melanopsin, as numerous studies have revealed that this pigment has *λ*_max_ around 480 nm in its native environment. Cells expressing mouse Opn4 showed robust irradiance and wavelength-dependent differences in aequorin luminescence in the presence of 11-*cis* retinal. The EC50 values of sigmoidal dose response curves were converted to a relative sensitivity and fitted with an opsin : retinaldehyde pigment template function [38]. The optimal *λ*_max_ for the template was determined by least squares as 484 nm (figure 3*a*), although templates with *λ*_max_ several nanometres either side of that figure showed nearly equivalent fit to these data (figure 3*b*).

Cells expressing human melanopsin similarly showed robust irradiance and wavelength-dependent differences in aequorin luminescence and irradiance response curves were constructed for eight wavelengths across the visible light spectrum (see the electronic supplementary material, figure S2). The relative sensitivity values were calculated and fitted as above. In the case of human Opn4, these data were well fit with templates corresponding to *λ*_max_ around 479 nm (figure 3*c,d*).

## 4. Discussion

We present the first detailed investigation of the spectral sensitivity and G-protein selectivity of human melanopsin. Our data indicate that, at least under heterologous expression in HEK293 cells, human melanopsin exhibits robust G_q__/11_ signalling, while exhibiting some ability to interact with G_i/o_, and that it has a spectral sensitivity (*λ*_max_ = 479 nm) very similar to that previously reported for other mammalian species.

### (a) G-protein selectivity of human melanopsin

Human melanopsin has all of the structural motifs necessary to activate G-protein signalling cascades [39]. Our strategy for exploring which class(es) of G protein it can couple to has been to assess its ability to modulate second messenger systems when heterologously expressed in HEK293 cells. Employing assays capable of detecting the activity of the three major Gα family signalling cascades and comparing responses with those elicited by opsins known to strongly activate G_i/o_ and G_s_ cascades has enabled us to test the hypotheses that human melanopsin can drive light-dependent activation of G_s_, G_i_ and G_q_ family cascades.

HEK293 cells provide a good test bed for G_s_ signalling, with light driving high amplitude increases in cAMP (as revealed by glosensor luminescence) in cells expressing JellyOp (an opsin known to be G_s_ coupled). Nevertheless, light induced no cAMP reporter increase in human melanopsin-expressing cells. This finding could simply reflect poor melanopsin expression in this system, but as human melanopsin did drive other signalling events, it rather indicates that this pigment has little if any coupling to the G_s_ cascade.

Human melanopsin was associated with a small but consistent reduction in cAMP following light exposure. This response could, in part, be secondary to changes in intracellular calcium, via calcium-sensitive adenylyl cyclases, or be driven by a Gα-independent mechanism such as free G*_βγ_* subunits [40]. However, its sensitivity to pertussis toxin indicates a contribution of direct G_i/o_ coupling for human melanopsin. This is consistent with a previous report that mouse melanopsin can activate a G_i_ family member (G_t_) when purified *in vitro* [25].

The most significant melanopsin light response was observed in cells expressing the aequorin reporter, indicating that melanopsin induces a strong increase in intracellular calcium. Such a response could be secondary to G_i/o_, or in particular G_s_, activity but this seems unlikely because human melanopsin did not drive significant G_s_ activity in these cells, while the calcium response was not pertussis toxin sensitive. Although we did not examine G_12/13_ coupling of melanopsin in this study, we also consider this an unlikely origin for the calcium response. G_12/13_ proteins primarily activate the RhoA signalling pathway and, while they have been associated with alterations in calcium and cAMP levels, this occurs synergistically with G_s_, G_i_ and/or G_q__/11_ responses [41–43]. It therefore is unlikely that G_12/13_ activation alone could be responsible for the melanopsin-dependent second messenger changes we have observed here. The most parsimonious explanation is that this fast calcium response is a direct consequence of activation of a G_q_ family α subunit, a conclusion consistent with previous work (see [44] for review).

Based upon the relative amplitude of calcium and cAMP responses, it is tempting to conclude that human melanopsin shows more efficient G_q__/11_ than G_i/o_ coupling. However, as these assays may have different sensitivities, we are unable to draw that conclusion with certainty. Moreover, as G-protein selectivity could be cell-type-dependent, it is safest simply to conclude that both G_q__/11_ than G_i/o_ are viable origins for the human melanopsin light response.

### (b) Second messenger signalling in melanopsins from other species

Aequorin responses driven by melanopsins from amphioxus, mouse and chicken were all equivalent to those elicited by the human pigment, consistent with the view that all induce G_q__/11_ signalling (figure 2*a,b*). The small cAMP reduction induced by human melanopsin indicative of G_i/o_ coupling was also observed in mouse and amphioxus melanopsin. Interestingly, however, the two chicken melanopsins drove a light-dependent increase in cAMP to different levels. We conclude this reflects G_s_ activation in these cells rather than a secondary effect of calcium mobilization as the calcium reporter levels were equivalent with those in human melanopsin-expressing cells that show no increase in cAMP. It would be interesting to investigate whether these differences in cyclic nucleotide signals of two different melanopsin genes within the same species are the cause of variations in electrophysiological activity previously described [45].

### (c) Melanopsin spectral sensitivity

The strategy used here to describe the spectral sensitivity of human melanopsin has several advantages over alternative methods for defining this feature of visual pigments. The highest resolution descriptions of photopigment spectral sensitivity have traditionally come from direct measures of light absorbance. However, these require high pigment concentrations. In the case of many conventional visual photoreceptors, specialized cellular compartments (e.g. rod outer segments) meet this criterion, allowing microspectrophotometric measures of pigment absorption in its native environment. In other cases, *in vitro* samples with sufficiently high pigment density for spectrophotometry have been produced by purifying opsin from retinal homogenates, or following heterologous expression in cell culture. Getting reconstituted pigment *in vitro* has however proved challenging for many opsins and, in the case of mammalian melanopsins has produced absorbance spectra which are inconsistent from study to study and do not always provide a good approximation of physiological estimates of ipRGC spectral sensitivity [25–27,46].

An alternative to direct assessments of pigment absorption is to construct an action spectrum, based upon measuring the spectral sensitivity of physiological responses driven by the pigment in question. The signal amplification inherent in G-protein pathways means that it is possible to define action spectra for photoreceptors lacking pigment concentrations suitable for microspectrophotometry (as is the case for ipRGCs). Moreover, provided certain criteria are met, the physiological response recorded can be many steps removed from photoreceptor, allowing action spectra to be constructed even when it is not possible to record the photoreceptor response directly. These advantages of action spectra are traditionally offset by a couple of significant disadvantages. Firstly, it is often difficult to measure physiological/behavioural responses evoked by the photoreceptor of interest. In the case of human melanopsin, this is because it is hard to exclude rod and/or cone influence on any downstream physiological response. In other cases, it could be because the species itself is not suitable for laboratory studies. The strategy adopted here of recording the physiological response to the pigment expressed in cell culture overcomes these limitations. The second limitation of the action spectrum approach is that it typically defines spectral sensitivity with lower resolution than more direct measurements of light absorption. Action spectra are built from well-defined irradiance response curves to monochromatic light, each of which requires a large number of physiological recordings. This places a practical limit on the number of wavelengths sampled. Our use of luminescent reporters to assay the photoreceptor light response mitigates this limitation to some extent by making it relatively easy to record a large number of physiological responses. The assays used here work in a multiwell plate format allowing the individual recordings upon which each action spectrum is based to be collected relatively quickly.

A concern with any strategy based upon heterologous expression is that the protein's behaviour may be affected by the non-native environment. We have controlled for this possibility by using the same strategy to define the spectral sensitivity of mouse melanopsin. Our estimate for its *λ*_max_ (484 nm) fits previous data for this pigment expressed in HEK293 cells and *Xenopus* oocytes and independent estimates based upon the spectral sensitivity of mice lacking rods and cones [24,29,47]. We are therefore confident that the HEK293 expression system provides the best currently available method of assessing the spectral sensitivity of human melanopsin (and other difficult to study pigments).

We find that the human melanopsin action spectrum can be closely fit by the predicted absorbance spectrum of an opsin : vitamin A1 photopigment with a *λ*_max_ of 479 nm (figure 3*b*). This represents the first detailed assessment of human melanopsin's spectral sensitivity (although a preliminary assessment indicated that this pigment may have a different *λ*_max_ in Neuro2A cells [46]). The estimate of *λ*_max_ 479 nm fits well with direct measurements of ipRGC spectral sensitivity following loss of rod and cone input in monkey, rat and mouse [7,29,33]. It is also consistent with action spectra for two physiological responses in humans that could be dominated by melanopsin photoreception (sustained pupil constriction and an aspect of visual adaptation) [32,33]. Action spectra for melatonin suppression in humans (a response downstream of ipRGCs in other species [48,49]) have *λ*_max_ some 20 nm shorter than the melanopsin action spectrum presented here [30]. This could reflect an influence of short wavelength cones on the melatonin response; however, both studies reported univariant irradiance response curves across the wavelengths tested [48,49] indicative of reliance on a single pigment. An alternative explanation is that the discrepancy arises mainly from differences in methods of analysis [50].

Melanopsin photoreception has now been implicated in a wide array of physiological responses as well as in aspects of perceptual vision. There is thus great interest in the extent to which current lighting design engages this new photoreceptor and the potential for careful regulation of light exposure to improve human health and well-being. A critical first step in this process is the creation of methods of measuring light that predict its impact on melanopsin. This requires an acceptance of the relative sensitivity of this photopigment to different wavelengths. We have recently proposed a new photometric measure (‘melanopic illuminance’) for ambient illumination, which accurately predicts the response of mouse melanopsin to a variety of lighting conditions [51]. The method for calculating melanopic illuminance described in Enezi *et al.* [51] assumes that melanopsin has *λ*_max_ around 480 nm. That seems well established for mice, and we believe the data presented here adds to the evidence that this is also true for humans.
